# Neurobrucellosis: laboratory features, clinical characteristics, antibiotic treatment, and clinical outcomes of 21 patients

**DOI:** 10.1186/s12879-024-09308-x

**Published:** 2024-05-10

**Authors:** Wei Zhuang, Tao He, Jianyekai Tuerheng, Guang He, Bao-Li Wang, Yi-Heng Yang, Lan Zhang, Xian-Zhe Dong, Sheng-Yan Xi

**Affiliations:** 1 Department of Pharmacy, Xuanwu Hospital of Capital Medical University, National Gerontic Disease Clinical Research Center, Beijing, China; 2 Department of Pharmacy, Eye Hospital China Academy of Chinese Medical Sciences, Beijing, China; 3 Clinical Medicine of Traditional Chinese and Western Medicine, Heilongjiang University of Chinese Medicine, Harbin, China; 4 Department of Pharmacy, Peking University Third Hospital, Beijing, China; 5 Department of Traditional Chinese Medicine, School of Medicine, Xiang’an Hospital, Xiamen University, Xiamen, China

**Keywords:** Neurobrucellosis, Clinical symptom, Supplementary examination, Treatment plan, Retrospective research

## Abstract

**Background:**

Neurobrucellosis (NB) is a rare and serious complication of brucellosis. Its clinical manifestations vary, with no obvious specificity. At present, there is no clear clinical diagnosis or treatment for reference. In this study, we retrospectively analyzed the clinical data for 21 patients with NB to provide reference data for its further study.

**Methods:**

We analyzed the epidemiological and clinical manifestations, laboratory tests, imaging examinations, cerebrospinal fluid, and treatment plans of 21 patients diagnosed with NB in the Department of Neurology, Xuanwu Hospital, Capital Medical University Beijing, China.

**Results:**

The ages of the patients ranged from 15 to 60 years old (mean age 40.1 ± 13.33 years), the male: female ratio was 4.25:1. Thirteen patients had a history of animal (sheep, cattle) contact, three had no history of animal contact, and the contact status of four was unknown. Brucella can invade various systems of the body and show multi-system symptoms, the main general manifestations were fever (66.67%), fatigue (57.14%) and functional urination or defecation disturbance (42.86%). The main nervous system manifestations were limb weakness (52.38%) and hearing loss (47.62%).The main positive signs of the nervous system included positive pathological signs (71.43%), sensory abnormalities (52.38%), limb paralysis (42.86%). Nervous system lesions mainly included spinal cord damage (66.67%), cranial nerve involvement (61.90%), central demyelination (28.57%) and meningitis (28.57%). In patients with cranial nerve involvement, 69.23% of auditory nerve, 15.38% of optic nerve and 15.38% of oculomotor nerve were involved. The blood of eight patients was cultured for *Brucella*, and three (37.5%) cultures were positive and five (63.5%) negative. The cerebrospinal fluid (CSF) of eight patients was cultured for *Brucella*, and two (25.00%) cultures were positive and six (75.00%) negative. Nineteen of the patients underwent a serum agglutination test (SAT), 18 (94.74%) of whom were positive and one (5.26%) of whom were negative. A biochemical analysis of the CSF was performed in 21 patients, and the results were all abnormal. Nineteen patients underwent magnetic resonance imaging (MRI). Twenty-one patients were treated with doxycycline and/or rifampicin, combined with ceftriaxone, quinolone, aminoglycoside, or minocycline. After hospitalization, 15 patients improved (71.43%), two patients did not recover, and the status of four patients was unknown.

**Conclusions:**

The clinical manifestations, CSF parameters, and neurological imaging data for patients with NB show no significant specificity or correlations. When patients with unexplained neurological symptoms accompanied by fever, fatigue, and other systemic manifestations in a brucellosis epidemic area or with a history of contact with cattle, sheep, animals, or raw food are encountered in clinical practice, the possibility of NB should be considered. Treatment is based on the principles of an early, combined, and long course of treatment, and the general prognosis is good.

## Introduction

*Brucella* is a genus of Gram-negative bacteria that settle in cells and are commonly classified into sheep-type and bovine-type bacteria. The zoonosis caused by *Brucella* is called ‘brucellosis’, which is also known as ‘wavy fever’, ‘Mediterranean fever’, or ‘Malta fever’. It is an animal-specific infectious disease involving multiple systems, and is one of the commonest zoonoses in the world, with more than 500,000 new cases annually. It is prevalent in the Mediterranean Basin and countries in the Middle East, including Syria, Iraq, Iran, etc [1]... In China, it is mainly prevalent in pastoral areas, such as Inner Mongolia, Jilin, Heilongjiang, a Xinjiang, and Tibet, although cases occur in other provinces [2]. It is transmitted to humans through contact with infected animals (cattle or sheep) or the consumption of uncooked meat products or unpasteurized dairy products. The clinical manifestations in these patients include fever, fatigue, weakness, hidrosis, joint pain, reduced appetite, headache, muscle pain, low back pain, hepatosplenomegaly, and arthritis [3]. Neurobrucellosis (NB) is a rare complication of brucellosis, and its incidence in brucellosis patients varies between studies, but is usually < 10% [4–6]. *Brucella* enters the blood through the reticuloendothelial system of the human body and causes bacteremia, then invading meninges with high affinity. When the host immunity declines, the bacteria proliferate, after which they invade other structures of the nervous system through the damaged blood–brain barrier, resulting in various clinical symptoms, such as headache, limb weakness, hearing loss, meningitis, meningoencephalitis, myelitis, myelopathy, cranial neuritis, mental abnormalities, and vascular disturbances [7]. Because the clinical symptoms of NB have no clear specificity, many of the laboratory tests usually used to diagnose brucellosis often produce negative results, so there is no clear clinical diagnostic standard or treatment method for NB at present. This leads to a low diagnosis rate and a high misdiagnosis rate in the clinical context, and it is difficult to distinguish NB from many other diseases of the nervous system, including tuberculous meningitis and multiple sclerosis [8]. This necessarily poses great challenges for clinicians.

To improve the understanding of NB among clinical workers and to draw attention to it, in this study, we retrospectively analyzed the clinical data of 21 patients with NB, including its epidemiology, clinical manifestations, laboratory tests, imaging results, cerebrospinal fluid (CSF) parameters, and treatment plans, to provide reference data for further research into NB.

## Methods

From January 2014 to August 2020, 21 patients diagnosed with NB were admitted to the Department of Neurology, Xuanwu Hospital, Capital Medical University (Beijing, China), including 17 males and 4 females, aged 15–60 years (mean age, 40.1 ± 13.33 years). All the 21 patients met all the following criteria These were [9–11] (1) clinical features consistent with known NB and an epidemiological correlation; (2) isolation of Brucella from blood or CSF; and/or positive serum agglutination test (SAT)(titres _> 1/160);and presence of anti-Brucella antibodies in CSF. (3) changes in the CSF, the increased WBC count, increased protein concentration, or reduced glucose concentration; (4) a more suitable alternative diagnosis could not be established. The basic information, epidemiological history, laboratory test results (including routine CSF parameters, CSF and serum *Brucella* cultures, CSF and serum *Brucella* serum agglutination tests, and imaging results), clinical symptom records, treatment plans, and prognoses of the 21 patients were collected and analyzed. The use of the above patient information in the study was approved by the Ethics Committee of the hospital, in one case, a 15-year-old patient also obtained consent from himself and his legal guardian.

## Results

### Basic information

The ages of the 21 patients ranged from 15 to 60 years; one (4.76%) was < 18 years old and 20 (95.24%) were 18–60 years old (mean age, 40.1 ± 13.33 years). The age of NB onset was spread within the range of 18–60 years. Seventeen males and four females were enrolled, so the male: female ratio was 4.25:1. Therefore, the incidence rate was higher in males than in females. Eleven patients (52.38%) were from Hebei, two (9.52%) each from Inner Mongolia, Beijing, Henan, and Heilongjiang, and one (4.76%) from Shanxi, and the origin of one patient (4.76%) was unknown. Therefore, most patients came from northern China. Six patients (28.57%) had a history of contact with sheep (meat), 4 (19.05%) with cattle (meat) and sheep (meat), 3 (14.29%) with cattle (meat), and one patient (4.76%) had brucellosis in his family. Three patients (14.29%) had no history of animal contact and the histories of four (19.05%) were unknown. The incidence of NB was closely related to contact with cattle (meat) and sheep (meat) animals (Table 1).

**Table 1:** Analysis of basic information of 21 patients with NB

Basic item		Number of cases (%)
Gender	Male	17(80.95)
	Female	4(19.05)
Age	< 18	1(4.76)
	18–60	20(95.24)
Residence	Hebei Province	11(52.38)
	Inner Mongolia Autonomous Region	2(9.52)
	Beijing	2(9.52)
	Henan Province	2(9.52)
	Heilongjiang Province	2(9.52)
	Shanxi Province	1(4.76)
	Unknown	1(4.76)
Epidemiological history	Contact history of sheep	6(28.57)
	Contact history of cattle and sheep	4(19.05)
	Unknown	4(19.05)
	Contact history of cattle	3(14.29)
	No contact history	3(14.29)
	Family members have suffered from brucellosis	1(4.76)

### Clinical characteristics

The general manifestations were fever (14 patients,66.67%), fatigue (12 patients,57.14%), functional urination or defecation disturbance (nine patients,42.86%), myalgia and weight loss (two patients each, 9.52%), breathing difficulty and joint pain (one patient each, 4.76%). The nervous system manifestations were limb weakness (11 patients,52.38%), hearing loss (10 patients, 47.62%), headache and ambiguity of consciousness (three patients each, 14.29%), blurred vision (two patients, 9.52%), spastic paralysis of both lower extremities and lethargy (one patient each, 4.76%).,. Nervous system symptoms included pathological signs (15 patients, 71.43%), sensory abnormalities (11 patients, 52.38%), limb paralysis (nine patients, 42.86%), ataxia (five patients, 23.81%), eye movement disorder (three patients, 14.29%), mental and behavioral abnormalities (two patients, 9.52%), and meningeal irritation signs (one patient, 4.76%). Nervous system lesions mainly included spinal cord damage (14 patients, 66.67%), cranial nerve involvement (13 patients, 61.90%), central demyelination (six patients, 28.57%), meningitis (six patients, 28.57%), encephalitis (five patients, 23.81%) and peripheral nerve demyelination (one patient, 4.76%). In patients with cranial nerve involvement, 69.23% of auditory nerve, 15.38% of optic nerve and 15.38% of oculomotor nerve were involved. These data indicate that the main clinical manifestations of NB are fever, fatigue, limb weakness, hearing loss, disturbed urination or defecation function, positive physical examination pathological signs, limb paralysis, and sensory abnormalities, usually involving the spinal cord, auditory nerve, and other parts of the nervous system (Table 2).

**Table 2:** Analysis of clinical characteristics of 21 patients with NB

The general manifestations								
Type		Fever	Fatigue	Functional urination or defecation disturbance	Myalgia	Weight loss	Breathing difficulty	Joint pain
Number of cases (%)		14 (66.67%)	12 (57.14%)	9 (42.86%)	2 (9.52%)	2 (9.52%)	1 (4.76%)	1 (4.76%)
The nervous system manifestations								
Type		Limb weakness	Hearing loss	Ambiguity of consciousness	Headache	Blurred vision	Spastic paralysis of both lower extremities	Lethargy
Number of cases (%)		11 (52.38%)	10 (47.62%)	3 (14.29%)	3 (14.29%)	2 (9.52%)	1 (4.76%)	1 (4.76%)
Positive signs of nervous system								
type		Positive pathological sign	Sensory abnormality	Limb paralysis	Ataxia	Eye movement disorder	Abnormal mental behavior	Positive meningeal irritation sign
Number of cases (%)		15 (71.43)	11 (52.38)	9 (42.86)	5 (23.81)	3 (14.29)	2 (9.52)	1 (4.76)
Involved nervous system								
Type	Spinal cord damage	Central demyelination	Meningitis	Encephalitis	Peripheral nerve demyelination	Cranial nerve involvement (13,61.90%)		
						Auditory nerve	Optic nerve	Oculomotor nerve
Number of cases (%)	14 (66.67%)	6 (28.57%)	6 (28.57%)	5 (23.81%)	1 (4.76%)	9 (69.23%)	2 (15.38%)	2 (15.38%)

### Laboratory test results

(1) Bacterial culture examination: Of the eight *Brucella* blood cultures performed, three (37.50%) were positive and five (63.50%) were negative. Of the eight *Brucella* CSF cultures performed, two (25.00%) were positive and six (75.00%) were negative. (2) *Brucella* agglutination test: Of the 19 patients tested with SAT, 18 (94.74%) were positive and one (5.26%) were negative for *Brucella*. (3) CSF biochemical examination: All 21 patients who underwent a CSF biochemical examination showed an abnormal result. The CSF pressure was elevated in 8 patients (40.00%), the WBC count was elevated in 21 patients (100.00%), glucose in CSF to serum glucose ratio (%) was reduced in 21 patients (100.00%), IgG concentration and the protein concentration were elevated in 21 patients (100.00%), and the chlorine concentration was reduced in 14 patients (66.67%). (4) Imaging examination. Of the 19 patients examined with MRI, three showed no obvious abnormalities and 16 (84.21%) showed imaging abnormalities, which included white matter changes in 8 patients (42.11%), inflammatory changes in five patients (26.32%), vascular involvement in four patients (21.05%), and meningeal enhancement in three patients (15.79%). (Table 3).

**Table 3:** Analysis of laboratory test results of 21 patients with NB

Check item		Number of cases (%)	Cerebrospinal fluid		Number of cases (%)
Blood culture	Positive	3/8 (37.5)	Pressure (mmH_2_O)	Normal	12/20 (60.00)
	Negative	5/8 (63.5)		Elevate	8/20 (40.00) 1
	Not detected	13			
CSF culture	Positive	2/8 (25.00)	WBC (*10^6^/L)	< 100	12/21 (57.14)
	Negative	6/8 (75.00)		100–500	8/21 (38.10)
	Not detected	13		> 500	1/21(4.76)
Serum agglutination test	Positive	18/19 (94.74)	CSF/serum glucose (%)	<60%	21/21 (100.00)
	Negative	1/19 (5.26)	IgG(mg/dl)	Elevate	21/21 (100.00)
Imaging	White matter alteration	8/19 (42.11)	Protein concentration (mg/dl)	Elevate	21/21 (100.00)
	Inflammatory change	5/19(26.32)			
	Vascular involvement	4/19 (21.05)			
	No obvious abnormality	3/19 (15.79)	Chlorine concentration(mmol/L)	Reduce	14/21 (66.67)
	Meningeal enhancement	3/19 (15.79)		Normal	7/21 (33.33)
	Not detected	2			

Remarks: Reference values of cerebrospinal fluid biochemical examination: cerebrospinal fluid pressure 80–180 mmH2O, WBC < 10 × 10^6^/L, protein concentration 15–45 mg/dl, chlorine concentration 118-128mmol/L, IgG 0.48-5.86 mg/dl, CSF/ serum glucose 60–70%.

### Antibiotic treatments and clinical outcomes

Twenty-one patients were treated with doxycycline (0.1 g, twice a day) and/or rifampicin (0.45–0.6 g, once a day), combined with ceftriaxone (2 g, once a day), quinolones (e.g., 0.5 g of levofloxacin or 600 mg of ciprofloxacin, once a day), aminoglycosides (e.g., gentamicin 160,000 IU, once a day), or minocycline (100 mg, twice a day). According to the severity of the disease, 15 patients (71.43%) had improved or their CSF biochemical indices tended to normal or even completely normal after 2–27 days of hospitalization. However, two patients (9.52%) showed no obvious improvement and four patients (19.05%) had no follow-up treatment results (Table 4).

**Table 4:** Analysis of treatment plans and prognoses of 21 patients with NB

Program		Course of treatment in hospital (days)	Number of cases	Prognosis		
				Get better	Unhealed	Unknown
Dual therapy	Doxycycline 0.1 g bid + rifampicin 0.45 g qd	3–12	4	2	1	1
	Doxycycline 0.1 g bid + ceftriaxone 2 g qd	12	1	1		
	Doxycycline 0.1 g bid + gentamicin 160,000 iu qd	7	1	1		
Triple therapy	Doxycycline 0.1 g bid + rifampicin 0.45–0.6 g qd + ceftriaxone 2 g qd	2–22	8	6	1	1
Monotherapy + Dual therapy + Triple therapy	Doxycycline, streptomycin, levofloxacin, ceftriaxone, rifampicin, ciprofloxacin, gentamicin, minocycline, and multiple drug replacement regimens	12–27	7	4		2
Total			21	15	2	4

## Discussion

Brucellosis is the most common infectious animal disease throughout the world, and in China, it is mainly seen in Inner Mongolia, Shanxi, Heilongjiang, Xinjiang Uygur Autonomous Region, Hebei, Liaoning, Jilin, and Ningxia Hui Autonomous Region. Most of the patients in this study were from Hebei, Inner Mongolia, Heilongjiang, and Shanxi, consistent with other epidemiological studies [2]. However, four patients were from Beijing or Henan. With the development of tourism in pastoral areas and the migration of the labor force, the population mobility between pastoral areas and non-pastoral areas has increased greatly. Therefore, the incidence rate of brucellosis in non-pastoral areas shows an upward trend, as does the *Brucella* infection rate in the non-occupationally-exposed population [7]. The most common sources of human brucellosis are sheep and cattle with brucellosis [1]. Humans are infected by ingesting untreated milk or dairy products or by contacting infected animals, animal excreta, or meat, and the transmission routes include the lung, conjunctiva, skin, and digestive tract, since there are many pathogenic bacteria in raw milk, the risk of infection by bacteria is very high, so this factor was also considered in the epidemiological investigation, but the medical records included in the study did not have the habit of taking raw milk, so there were no relevant medical records, and the rest is consistent with the etiology demonstrated in this study. It is also noteworthy that of one exceptional patient in this study, had a family member suffering brucellosis and the cause of family member was consumption of mutton with brucellosis infection, which had nothing to do with other reasons such as consumption of raw milk or dairy products or sexual intercourse. Therefore, in any epidemiological investigation of patients suspected of *Brucella* infection, we should also ask whether there is a recent history of travel to a pastoral area, a family history of brucellosis, or a history of contact with unusual pathogenic animals.

The mechanism by which *Brucell*a invades the central nervous system is still unclear. It is generally believed that *Brucella* causes bacteremia after entering the blood through the reticuloendothelial system of the human body, and then invades the meninges with particular affinity. When the host immunity declines, *Brucella* begins to proliferate and invades other nervous system structures [12]. Recent studies have shown that Brucella induces tissue damage indirectly, most likely by activating the host’s innate immune response after recognizing the corresponding receptor by Brucella antigen. Lipoprotein is an important brucella antigen that induces a variety of innate immune cells to secrete pro-inflammatory cytokines. Brucella nucleic acids have also been shown to be involved in the induction of inflammation. Astrogliosis and reactive microgliosis are unique signs of central nervous system inflammation associated with NB. Dysregulation of astrocytes and microglia caused by brucella infection creates a microenvironment in the central nervous system in which secretion of pro-inflammatory mediators leads to instability of glial structures, damage to the blood-brain barrier, and neuronal death. The immune mechanism of astrocyte hyperplasia and reactive microgliosis induced by Brucella is through promoting the secretion of inflammatory cytokines IL-6, IL-1β, TNF-α, and chemokines CCL2 and CXCL1 [13]. The clinical manifestations of NB are varied, and the common clinical manifestations include fever, fatigue, myalgia, weight loss, splenomegalia, aphasia, vertigo, double vision, facial paralysis, hemiplegia, tremor, ataxia, and psychiatric symptoms [13, 14]. They also include meningitis, encephalitis, cerebrovascular diseases, subarachnoid haemorrhage, myelitis, radiculitis, cranial or peripheral nerve involvement, intracerebral and epidural abscess, demyelinating syndrome [11, 13]. The clinical manifestations observed in this study included fever, fatigue, limb weakness, hearing loss, defecation disorder, headache, unconsciousness, myalgia, weight loss, spastic paralysis of both lower limbs, hyperhidrosis, lethargy, breathing difficulties, and joint pain, among which systemic fever, fatigue and disturbed urination or defecation functions were common, together with nervous system symptoms such as limb weakness, hearing loss, unconsciousness and headache. Clinical examination often observed positive pathological signs, paresthesia, ataxia, eye movement disorder, and meningeal irritation signs, and the rate of meningeal irritation signs (1/21) was 4.76%, consistent with that reported in the literature (< 50%) [13]. The most common clinical presentations of NB include meningitis, meningoencephalitis, myelitis, brain abscess, epidural abscess, radiculitis, cranial neuritis, demyelinating disease, and vascular disease, among which meningitis and meningoencephalitis account for 50% [12]. Meningitis, the most typical clinical manifestation of NB, leads to irreversible central nervous system damage and is associated with neuronal damage. Overactivation of microglia leads to neurotoxicity and is one of the factors of neuronal injury in the central nervous system. Activated microglia can also secrete a variety of soluble mediators; however, most of the mediators produced are proinflammatory and neurotoxic [11]. The cause of central nervous system inflammation and vasculopathy caused by NB may be the invasion of the central nervous system by Brucella, which interacts with astrocytes and microglia and induces the secretion of TNF-α, IL-1β and IL-6 by astrocytes and microglia. The induced inflammatory response will promote the activation of cerebral microvascular endothelium, the increase of vascular fluidity and the infiltration of immune cells [11]. NB can involve one or more cranial nerves. Auditory nerve involvement is most common, and is generally considered to be caused by the involvement of the central auditory nerve pathway. The second most commonly involved nerve is the abducens nerve, which may be vulnerable to both direct and indirect injury because it has the longest intracranial run. The third most commonly involved nerve is the facial nerve, and NB can also damage the optic nerve [4]. In this study, the most frequently involved neurological lesions included spinal cord lesions, cranial nerve involvement, central demyelination, meningitis, encephalitis and peripheral nerve demyelination, among which spinal cord lesions (14/21) occurred in 66.67% of patients, cranial nerve involvement (13/21) in 61.90%, central demyelination (6/21) in 28.57%, meningitis (6/21) in 28.57%, encephalitis (5/21) in 23.81% and peripheral nerve demyelination (1/21) in 4.76%, and in patients with cranial nerve involvement, 69.23% of auditory nerve, 15.38% of optic nerve and 15.38% of oculomotor nerve were involved. It is noteworthy that the common clinical manifestations of NB rarely include diurnal disorders, although the incidence of urination or defecation functional disturbance (9/21) among the patients in this study was 42.86%, which may have been caused by spinal cord involvement.

The most reliable criterion for the diagnosis of NB is a positive bacterial culture, but the positive rate of this method is low, and most patients also require a serum or CSF agglutination test to confirm their diagnosis [15]. Blood cultures of *Brucella* were performed in eight patients, of which three were positive (37.50%) and five were negative (63.50%). CSF cultures of *Brucella* were performed in eight patients, of which two were positive (25.00%) and six were negative (75.00%). Of the 19 patients tested with SAT, 18 were positive (94.74%) and one was negative (5.26%). The positive rate of SAT was higher. The results of the CSF biochemical analysis were abnormal in all 21 patients tested. The CSF pressure was increased in 8 patients (40.00%), the WBC count was elevated in 21 patients (100.00%), the glucose in CSF to serum glucose ratio (%) was reduced in 21 patients (100.00%), IgG concentration and the protein concentration were elevated in 21 patients (100.00%), and the chlorine concentration was reduced in 14 patients (66.67%). Analysis of the CSF biochemical results showed that the CSF of NB patients was altered, but there was no obvious specificity, which was usually WBC, IgG concentration and protein concentration increased, glucose in CSF to serum glucose ratio (%) and chlorine concentration decreased, and may be accompanied by increased CSF pressure to different degrees. The changing trends in the WBC count, protein concentration, and glucose concentration are consistent with reports in the literature [16, 17], but the incidence of reduced glucose concentration (66.67%) was higher than previously reported (1/3, 33.33%) [18]. Leukocytosis in CSF may be due to Brucella activation of glial cells in the blood-brain barrier, and Brucella interaction with innate immunity in the central nervous system leads to increased transfer of phagocytic cells to the brain parenchyma [11]. In 19 patients, MRI showed no obvious abnormalities in three patients but abnormalities in 16 patients. The main manifestations were white matter changes (42.11%), inflammatory changes (26.32%), vascular involvement (21.05%), and meningeal enhancement (15.79%). The first three manifestations have been reported in other research [3, 19]. In the present study, the lesions involved in the white matter were mainly concentrated around the lateral ventricle, temporal lobe, parietal lobe, cerebellar middle foot, and pons, which showed abnormal signals in the white matter. At present, the specific causes and mechanisms of abnormal white matter are unclear. It is thought that *Brucella* infection may activate the immune system, leading to demyelinating changes in the white matter [3]. Meningeal lesions were concentrated in the temporal lobe, frontal part, and anterior skull base.

Because *Brucella* usually enters the cell cytoplasm for its survival and reproduction and most antibacterial drugs have weak penetration ability and must pass the blood–brain barrier, it is difficult for ordinary antibacterial drugs to achieve a satisfactory curative effect. Therefore, antibacterial drugs with good fat solubility and strong intracellular and central nervous system penetration should be selected for the treatment of NB. Because it is difficult to kill intracellular *Brucella* with a short course of antibacterial treatment, a long course of treatment and combined medications are recommended [7]. Many studies have recommended antibiotics with good curative effects on NB, including doxycycline, rifampicin, streptomycin, compound sulfamethoxazole, and third-generation cephalosporins, such as ceftriaxone or ciprofloxacin [11, 20, 21], and rifampicin, doxycycline, and ceftriaxone are considered standard drugs for NB [20]. In the present study, 21 patients were treated with doxycycline and/or rifampicin, combined with ceftriaxone, quinolones, aminoglycosamines, or minocycline. After hospitalization, 15 patients improved, two patients did not recover, and the status of four patients was unknown. The medication regimens were consistent with the medications recommended in the literature [11, 20]. The two patients who did not recover were treated in hospital for 2–3 days, which we consider a short course of treatment. Although the best course of treatment for NB is still uncertain, the results of this study are consistent with those of other reported studies [4], and suggest that patients treated early with combined medications have a good prognosis.

In other cases reported on NB [22–24], the clinical characters of NB were analyzed from 1 to 3 aspects including clinical manifestations, serum/cerebrospinal fluid agglutination test, imaging and cerebrospinal fluid laboratory examination. However, only case reports and retrospective clinical studies involving less than 13 patients were reported, and the results were similar to those of this study. However, due to the small number of cases included in the study, the characteristics of a single case are mainly introduced, and the comparison cannot be made from probability. A study to assess the prevalence of imaging abnormalities in patients with NB included 263 adult patients at 26 referral centers [25], but only the imaging abnormalities in patients with NB were analyzed, and the results showed that 118 (45%) patients with NB had abnormal neuroimaging findings. The main manifestations were inflammation (95.76%) > vascular involvement (66.95%) > hydrocephalus/cerebral edema (50.85%) > white matter involvement (33.05%). In this study, the 21 cases with abnormal imaging accounted for 78.95%, which was significantly higher than the above studies, including white matter changes (42.11%) > inflammatory changes (26.32%) > vascular involvement (21.05%) > meninges enhancement (15.79%). The reason for the different proportion of abnormal manifestations may be related to the number of included medical records, which need to be further confirmed by multi-center and big data research. Compared with the above existing clinical studies, the number of cases included in this study is larger, and the analysis and comparison from multiple aspects such as epidemiology, clinical manifestations, criteria for inclusion of cases, imaging and laboratory examination of cerebrospinal fluid, can provide a more comprehensive basis for the subsequent diagnosis of NB patients.

However, some limitations should be noted. First, the small number of cases. When the sample size is too small, the shortcoming is less of cases and only few of the culture positive to derive very valid information. Second, this article is a retrospective case analysis study, and there may be some uncontrollable factors in the implementation process, such as inconsistent detection methods, specimen inspection, or NB-related necessary examinations are not carried out, so it is necessary to carry out prospective clinical trials.

## Conclusion

Brucellosis has always been a common and major health problem in developing countries, and NB is one of its serious complications. Although the incidence rate is usually < 10%, its early diagnosis and treatment are very important. In this study, we recorded and analyzed the clinical manifestations, laboratory and imaging results, and therapeutic outcomes of 21 patients with NB, to provide reference data for the diagnosis and treatment of this disease. Because NB is rare in the clinical context, there is no effective diagnostic “gold standard”, and the clinical manifestations, CSF parameters, and imaging changes lack specificity, which make its diagnosis very difficult. Therefore, when patients present with nervous system dysfunction accompanied by fever and fatigue, or have a contact history with animals and animal products, the possibility of NB should be considered, and empirical treatment for NB should be given if necessary. It is challenging to develop a highly specific and highly sensitive diagnostic regimen for NB, and the development of such a test will be the focus of research into the diagnosis and treatment of NB. At present, there is no consistent treatment plan for NB. Although the reports of NB have increased in recent years, most results obtained by researchers are observational, and the number of cases is still small. Therefore, multicenter clinical trials of NB are required.

## Appendix

**Table:** Attached: Detailed examination information of 21 patients

Patients	Clinical manifestations	Positive signs of nervous system	Involved nervous system	Affected cranial nerves	Imaging examination	Cerebrospinal fluid (CSF)						Blood/CSF culture	Serum agglutination test (SAT)
						Pressure (mmH_2_O)	WBC (×10^6^/L)	Protein (mg/dl)	Chlorine (mmol/L)	IgG (mg/dl)	CSF/serum glucose (%)		
1	Fever, fatigue, hearing loss, weakness of both lower limbs and urination and defecation function disturbance	Paralysis of limbs, positive pathological signs and abnormal sensation	Demyelination, spinal cord damage	8	Head MRI showed abnormal signals in the white matter around the anterior horn of the lateral ventricle on both sides. Cervical spinal cord MRI showed cervical 2-4-disc degeneration and cervical 3-5-disc herniation.	150	40	130	107	26.20	26	——	Positive
2	Fever, hearing loss	Positive pathological signs and abnormal sensation	Meningitis, encephalitis	8	No significant abnormalities were found in cervical and thoracic pulps. Head MRI showed abnormal white matter signals in the left temporal and parietal cortex. T2, Flair, DWI showed high signals.	210	76	136	104	35.30	25	——	Positive
3	Fever, headache, trance	Abnormal mental behavior	Meningitis, encephalitis	-	MRI of the head showed abnormal signals in the left frontal convex surface and right lateral fissure with bilateral temporal lobe meningeal enhancement.	180	599	301	104	155.00	36	——	Positive
4	Fatigue, hearing loss, spastic paralysis of both lower limbs	Positive pathological signs, ataxia	Demyelination, spinal cord damage	8	MRI of the head showed abnormal signal under the cortex of the left frontal parietal lobe and the right parietal lobe. Considering the possibility of demyelination, there was a small amount of subdural effusion in both the frontal and left temporal parts.	140	78	240	108	66.40	19	——	Positive
5	Blurred vision	Eye movement disorder	Meningitis, encephalitis, central demyelination	2	MRI of the head showed obvious enhancement of meninges on the left side of clivus, bilateral forehead, and anterior skull base, and abnormal enhancement shadows in bilateral optic nerve sheath, left hypoglossal nerve and jugular fossa, so it is possible to consider inflammatory changes.	215	48	131	108	67.30	28	——	Positive
6	Fever, joint pain, fatigue, limb weakness and urination and defecation function disturbance	Paralysis of limbs, positive pathological signs and abnormal sensation	Spinal cord injury	-	MRI of the thoracic spine and cervical spine showed no abnormality.	210	195	203	118	41.10	9	——	Positive
7	Fatigue, hearing loss, limb weakness, urination and defecation function disturbance	Paralysis of limbs, positive pathological signs and abnormal sensation	Central demyelination, spinal cord damage	8	Head MRI showed multiple abnormal signals in bilateral cerebellar middle pedunculus, pons, bilateral temporal lobes, and bilateral lateral ventricles.	190	62	115	121	14.10	39	——	Positive
8	Fever, weight loss, confusion	Positive pathological signs, abnormal sensation and ataxia	Central demyelination, spinal cord damage	2	MRI of the head showed an abnormal signal in the white matter of the right parietal lobe and an irregular flaky signal in the right temporal lobe with circular enhancement.	175	130	138	125	25.30	29	——	Positive
9	Fever, hearing loss	Ataxia	——	8	Head MRI showed lacunar cerebral infarction and white matter degeneration.	300	246	330	111	99.70	30	Positive(blood)	Positive
10	Blurred vision	Eye movement disorder	meningitis	3	MRI of the head showed no abnormality.	150	74	157	113	17.80	35	Negative	Positive
11	Fever, fatigue, hearing loss, limb weakness and urination and defecation function disturbance	Mental and behavioral abnormalities, limb paralysis, positive pathological signs, sensory abnormality, eye movement disorder and ataxia	Encephalitis, central demyelination, spinal cord damage	8	Head MRI showed demyelinating changes in white matter.	130	46	121	116	36.40	43	Negative	Positive
12	Myalgia	Positive pathological sign	spinal cord damage	-	Head MRI showed space-occupying lesions, complex cysts, and postoperative changes in the pineal region.	115	136	438	119	69.10	38	Negative	Positive
13	Fever, headache	Meningeal irritation sign, and pathological signs were positive	meningitis	3	MRI of the head showed multiple abnormal signals in bilateral frontal and parietal lobes, and ischemic lesions were considered first, except inflammatory changes.	310	270	120	111	12.90	33	Positive (CSF)	Negative
14	Fever, fatigue, hearing loss, limb weakness and urination and defecation function disturbance	Paralysis of limbs, positive pathological signs and abnormal sensation	spinal cord damage	8	——	170	173	466	95	238.00	29	——	Positive
15	Fever, fatigue, limb weakness	None	——	-	MRI of the head showed widening of the perivascular space in the left thalamus.	240	119	209	110	28.00	33	——	Positive
16	Fever	Sensory abnormality	Spinal cord injury, peripheral nerve demyelination	-	MRI of the cervical spinal cord showed abnormal signal in the spinal cord at C2/3 level.	180	91	169	110	62.30	25	——	Positive
17	Fatigue, limb weakness, urination and defecation function disturbance	Paralysis of limbs, positive pathological signs, abnormal sensation and ataxia	spinal cord damage	-	MRI of the head showed abnormal signal in the right lenticular nucleus, and the perivascular space may be.	175	171	197	114	37.60	21	Positive (blood)	Positive
18	Fever, headache, fatigue, hearing loss, limb weakness, and urination and defecation function disturbance	Paralysis of limbs with positive pathological signs	Meningitis, encephalitis, spinal cord damage	8	Head MRI showed multiple white matter degeneration and abnormal signal of gray matter in the brain, which may lead to encephalitis. Partially empty sella turcica. No obvious enhancement was found.	——	68	384	107	52.40	28	——	Positive
19	Fever, fatigue, hearing loss, and confusion	Positive pathological signs and abnormal sensation	spinal cord damage	8	——	145	14	83	119	27.80	59	——	Positive
20	Fever, fatigue, hearing loss, limb weakness and urination and defecation function disturbance	Paralysis of limbs, positive pathological signs and abnormal sensation	spinal cord damage	-	MRI of thoracic vertebrae showed Schumer nodules in thoracic vertebrae 10.	135	14	84	123	12.80	29	Positive (CSF)	——
21	Weakness of both lower limbs and urination and defecation function disturbance	Paralysis of limbs with positive pathological signs	spinal cord damage	-	MRI showed no abnormality in the thoracic spinal cord	205	9	160	126	21.70	29	Positive (blood)	——

## Data Availability

The datasets are not publicly available due to privacy or ethical restrictions but are available from the corresponding author on reasonable request.

## Abbreviations

NB: Neurobrucellosis
SAT: serum agglutination test
CSF: cerebrospinal fluid
WBC: white blood cell
MRI: magnetic resonance imaging
